# Supplementary material for: Spying on parahydrogen-induced polarization transfer using a half-tesla benchtop MRI and hyperpolarized imaging enabled by automation
Source: Nat Commun. 2023 Aug 8;14:4774. doi: 10.1038/s41467-023-40539-9 (PMC10409769; doi:10.1038/s41467-023-40539-9)
Supplement: Supplementary file 1 — Supplementary Information [file 41467_2023_40539_MOESM1_ESM.pdf]

Supplementary Information

Spying on parahydrogen-induced polarization transfer using a half-tesla benchtop MRI and hyperpolarized imaging enabled by automation

Frowin Ellermann[a]‡, Aidan Sirbu[b]‡†, Arne Brahms[c], Charbel Assaf[a], Rainer Herges[c], Jan-Bernd Hövener[a], Andrey N. Pravdivtsev[a]*

Contents

Supplementary note 1: Magnetic field drift....S-2
Supplementary note 2: OPE spin order transfer for ethyl acetate-*d6*....S-3
Supplementary note 3: OPE spin order transfer for ethyl cinnamate....S-4
Supplementary note 4: ^{1}H Signal enhancement....S-5
Supplementary note 5: ^{1}H NMR spectra and NMR parameters of EC, EA and EP....S-6
Supplementary note 6: ESOTHERIC SOT at high and low magnetic fields....S-15
Supplementary note 7: ^{13}C Polarization and enhancement....S-16

Supplementary note 1: Magnetic field drift

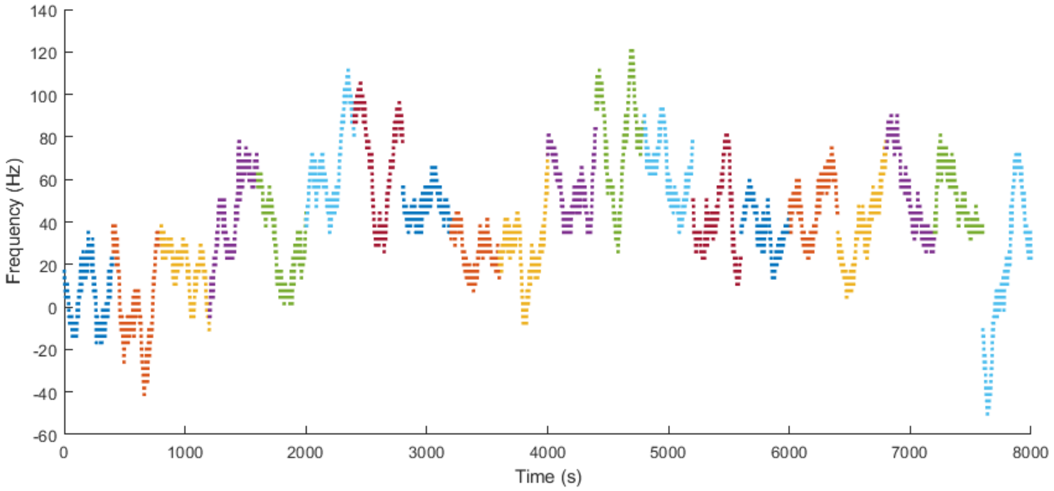

Supplementary Figure 1. Magnetic field drift. Resonance frequency offset as a function of time. ^{1}H Spectrum of acetone-h6 was measured as a function of time using a 5°-FID sequence and a repetition time of 0.1 s. The maximum of the peak after Fourier transformation was assigned to the resonance frequency. Standard deviation is about 80 Hz. Data were stored as chunks of 400 s, as indicated with colors.

Supplementary note 2: OPE spin order transfer for ethyl acetate-*d6*

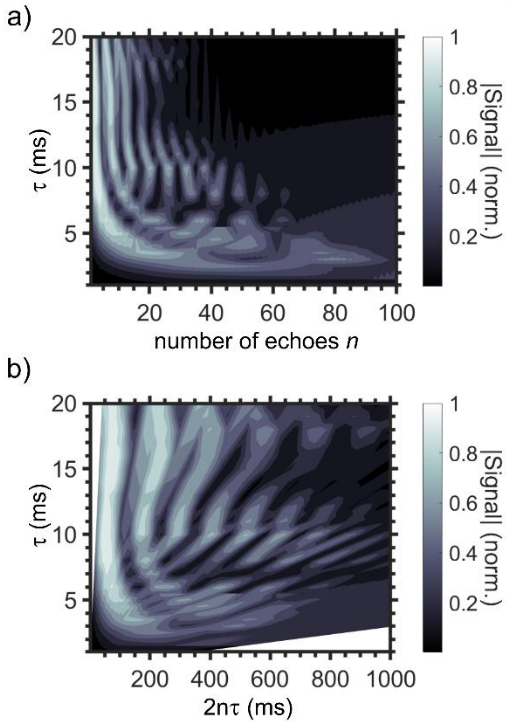

Supplementary Figure 2. Experimental observation of absolute signal in 45°-CPMG experiment for EA-*d6* at 0.55 T as a function of $\boldsymbol{\tau}$ and number of echoes $\boldsymbol{n}$ (a) or as a function of $\boldsymbol{\tau}$ and $\boldsymbol{2n\tau}$ (b). The samples were prepared by mixing [Rh] = 3 mM with 50 mM of the hydrogenation precursor (VA-*d6*) in acetone-*d6*. The pH_2 pressure was 15 bar. The signal decays due to relaxation, imperfect refocusing RF pulses, diffusion, and oscillation of the external magnetic field. The duration of the 90° and 180° pulses was 33 µs and 66 µs, respectively.

Supplementary note 3: OPE spin order transfer for ethyl cinnamate

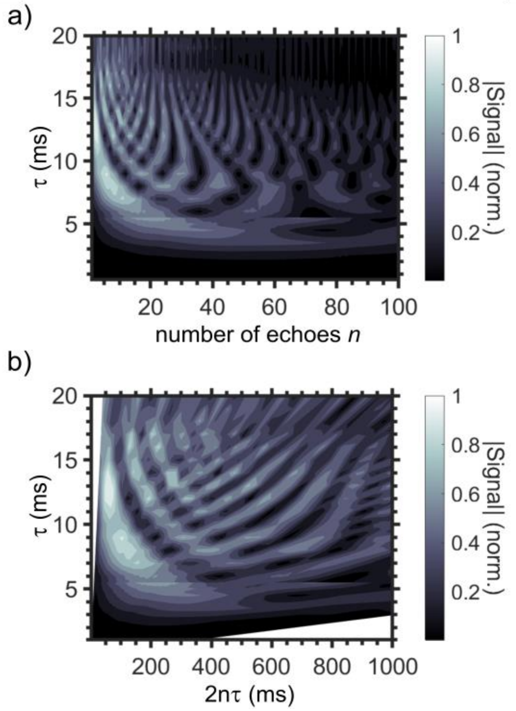

Supplementary Figure 3. Experimental observation of absolute signal in 45°-CPMG experiment for EC at 0.55 T as a function of $\boldsymbol{\tau}$ and number of echoes $\boldsymbol{n}$ (a) or as a function of $\boldsymbol{\tau}$ and $\boldsymbol{2n\tau}$ (b). The samples were prepared by mixing [Rh] = 3 mM with 50 mM of the hydrogenation precursor (EPh) in acetone-*d6*. The pH_2 pressure was 15 bar. The signal decays due to relaxation, imperfect refocusing RF pulses, diffusion, and oscillation of the external magnetic field. The duration of the 90° and 180° pulses was 33 µs and 66 µs, respectively.

Supplementary note 4: ^{1}H Signal enhancement

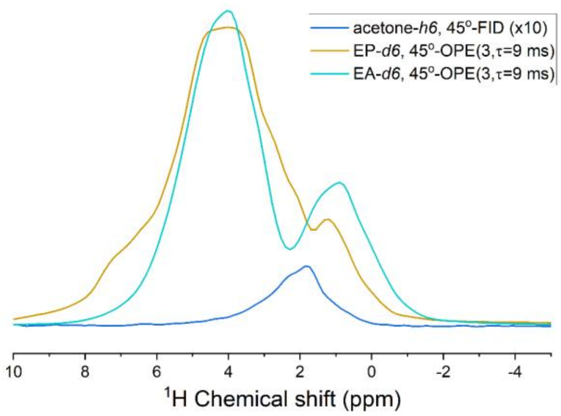

Supplementary Figure 4. Hyper- and thermally polarized spectra measured with the 0.55 T MRI.

EP-*d6* spectrum was obtained after 45 s of 92% pH_2 bubbling at 15 bar of the sample with [Rh] = 3 mM, [VP-d6] = 50 mM using 45°-OPE with τ = 9 ms and n=3; average polarization of protons was 14.4% that corresponds to average enhancement of 75250.

EA-*d6* spectrum was obtained after 20 s of 92% pH_2 bubbling at 15 bar of the sample with [Rh] = 3 mM, [VP-d6] = 50 mM using 45°-OPE with τ = 9 ms and n=3; average polarization of protons was 16.2% that corresponds to average enhancement of 84300.

Acetone-*h6* spectrum was obtained for the pure acetone-*h6* sample using 45°-FID; thermal polarization of protons was $\sim 1.918\times10^{-4}$%.

The acetone spectrum was multiplied by 10 for better visualization.

Signal enhancement and polarization were calculated as follows:

$$Enhancement\ = \frac{I(\text{PHIP})}{I(\text{Acetone})} \cdot \frac{6 \cdot [\text{Acetone}]}{2 \cdot [\text{Reagent}]}$$

$$P_{\text{th}} = \tanh\left(\frac{\gamma \cdot \hbar \cdot B_0}{2 \cdot k_B \cdot T}\right)$$

$$P\ =\ Enhancement \cdot P_{\text{th}}$$

Where $I(\text{PHIP})$ is the total integral for the PHIP spectrum, $I(Acetone)$ is the integral for the acetone-*h6* sample at thermal equilibrium, [X] are concentrations of acetone and a reagent (VP-*d6* or VA-*d6*, 50 mM), factors 6 and 2 are to take into account the number of protons, P_{th} is the value of thermal polarization, and P is polarization.

Supplementary note 5: ^{1}H NMR spectra and NMR parameters of EC, EA and EP

Ethyl cinnamate (EC)

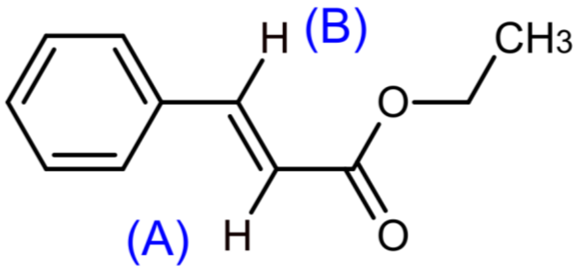

Supplementary Chart 1. Chemical structure of EC.

Supplementary Table 1. Chemical shifts and J coupling constants for EC in acetone were measured at 9.4 T and four temperatures.

T (K)	$\delta(H_A)$ (ppm)	$\delta(H_B)$ (ppm)	$\Delta\delta=\delta(H_A)-\delta(H_B)$ (ppm)	$J(H_A-H_B)$ (Hz)
293	5.9778	7.0026	-1.0248	12.76
303	5.9757	6.9977	-1.022	12.71
313	5.9735	6.9928	-1.0193	12.77
330	5.9711	6.9856	-1.0145	12.72

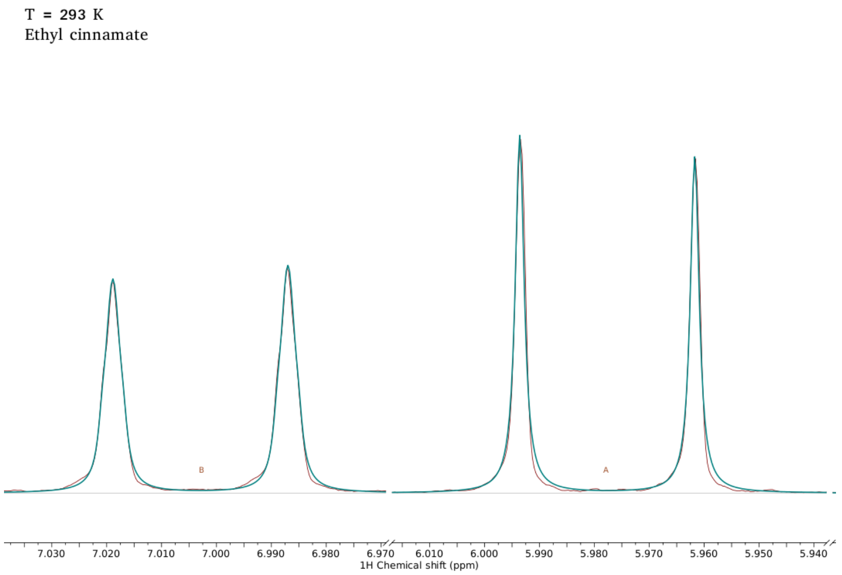

Supplementary Figure 5. Experimental and simulated ^{1}H NMR spectra of EC obtained at 9.4 T NMR. The samples were prepared as it is described in the text, hydrogenated using an automated system, and then placed into a 5 mm NMR tube for analysis. Simulation NMR parameters for EC: H_A (doublet, 5.9778 ppm, J(H_A-H_B) =12.76 Hz), H_B (doublet, 7.0026 ppm, J (H_B-two protons in the phenyl group) =0.56 Hz, J (H_B-one proton in the phenyl group) =0.3 Hz), line width was 0.78 Hz. The temperature of the sample was 293 K.

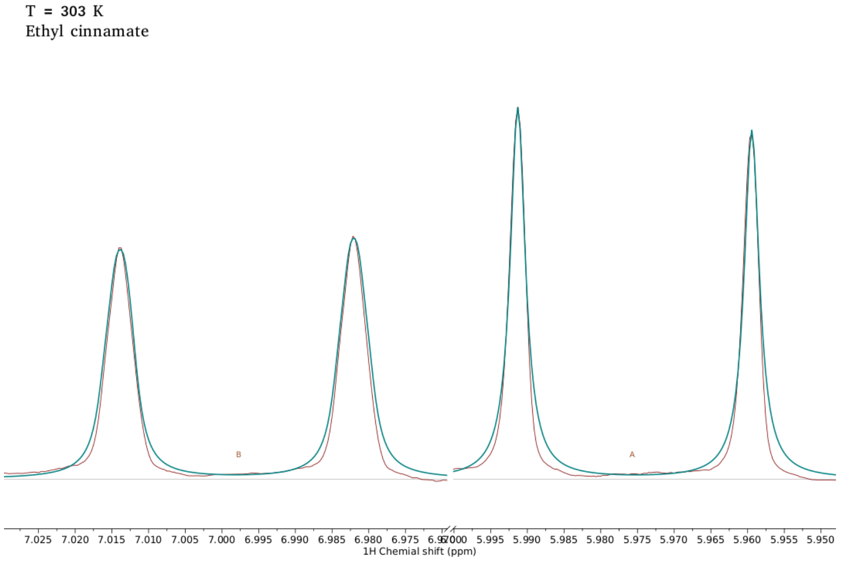

Supplementary Figure 6. Experimental and simulated ^{1}H NMR spectra of EC obtained at 9.4 T NMR. The samples were prepared as it is described in the text, hydrogenated using an automated system, and then placed into a 5 mm NMR tube for analysis. Simulation NMR parameters for EC: H_A (doublet, 5.9757 ppm, J(H_A-H_B) =12.77 Hz), H_B (doublet, 6.9977 ppm, J(H_B-two protons in the phenyl group)=0.62 Hz, J(H_B-one proton in the phenyl group)=0.275 Hz), line width was 1 Hz. The temperature of the sample was 303 K.

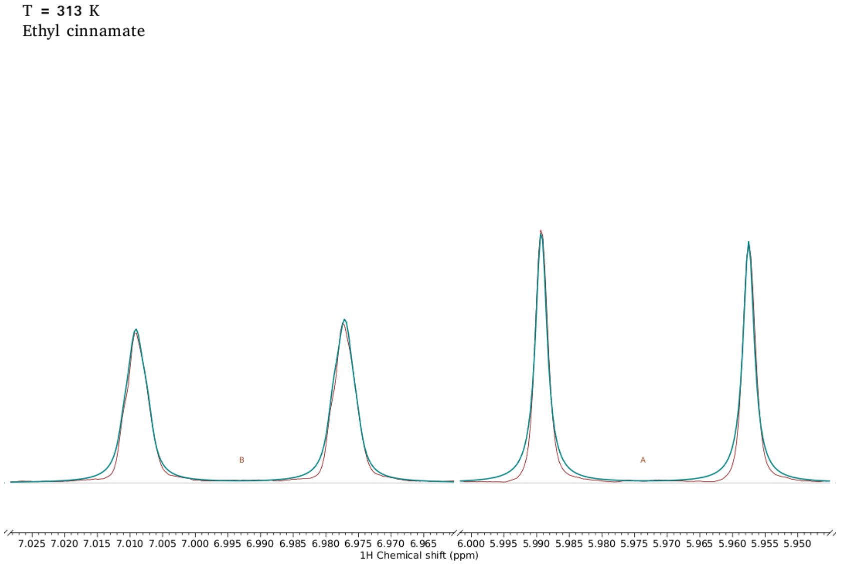

Supplementary Figure 7. Experimental and simulated ^{1}H NMR spectra of EC obtained at 9.4 T NMR. The samples were prepared as it is described in the text, hydrogenated using an automated system, and then placed into a 5 mm NMR tube for analysis. Simulation NMR parameters for EC: H_A (doublet, 5.9735 ppm, J(H_A-H_B) =12.71 Hz), H_B (doublet, 6.9928 ppm, J(H_B-two protons in the phenyl group)=0.59 Hz, J(H_B-one proton in the phenyl group)=0.32 Hz), line width was 0.83 Hz. The temperature of the sample was 313 K.

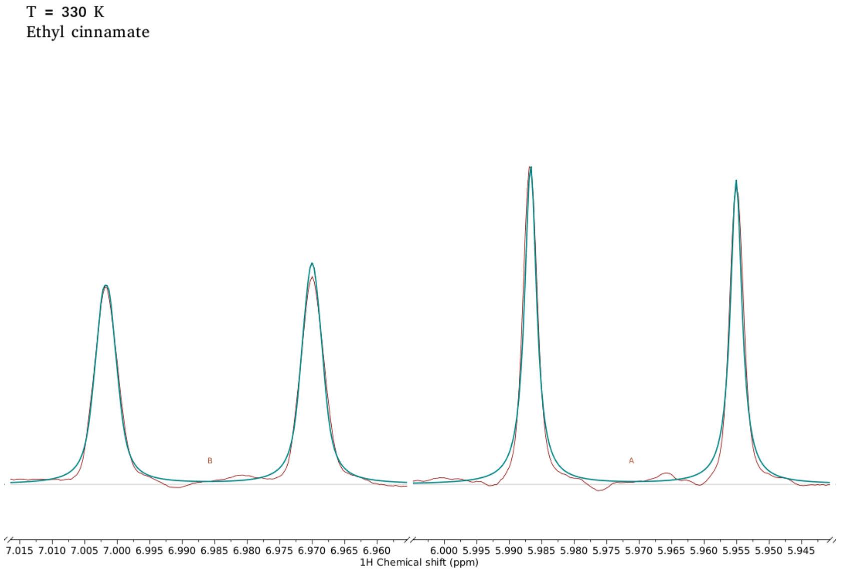

Supplementary Figure 8. Experimental and simulated ^{1}H NMR spectra of EC obtained at 9.4 T NMR. The samples were prepared as it is described in the text, hydrogenated using an automated system, and then placed into a 5 mm NMR tube for the analysis. Simulation NMR parameters for EC: H_A (doublet, 5.9711 ppm, J(H_A-H_B) =12.72 Hz), H_B (doublet, 6.9856 ppm, J(H_B-two protons in the phenyl group)=0.5 Hz, J(H_B-one proton in the phenyl group)=0.32 Hz), line width was 0.86 Hz. The temperature of the sample was 330 K.

Ethyl acetate (EA)

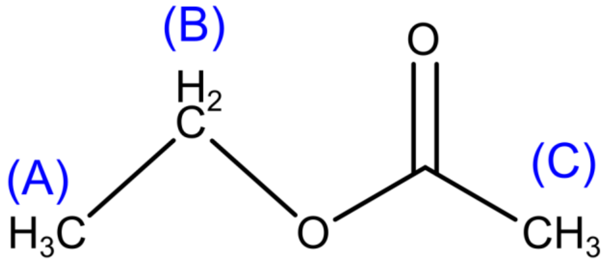

Supplementary Chart 2. Chemical structure of EA.

Supplementary Table 2. Chemical shifts and J coupling constants for EA in acetone were measured at 9.4 T and four temperatures.

T (K)	$\delta(H_A)$ (ppm)	$\delta(H_B)$ (ppm)	$\delta(H_C)$ (ppm)	$\Delta\delta=\delta(H_A)-\delta(H_B)$ (ppm)	$J(H_A-H_B)$ (Hz)	$J(H_B-H_C)$ (Hz)
293	1.2116	4.0654	1.9805	-2.8538	7.12	0.21
303	1.1980	4.0538	1.9626	-2.8558	7.12	0.2
313	1.2022	4.0598	1.9626	-2.8576	7.13	0.2
330	1.2091	4.0696	1.9626	-2.8605	7.12	0.23

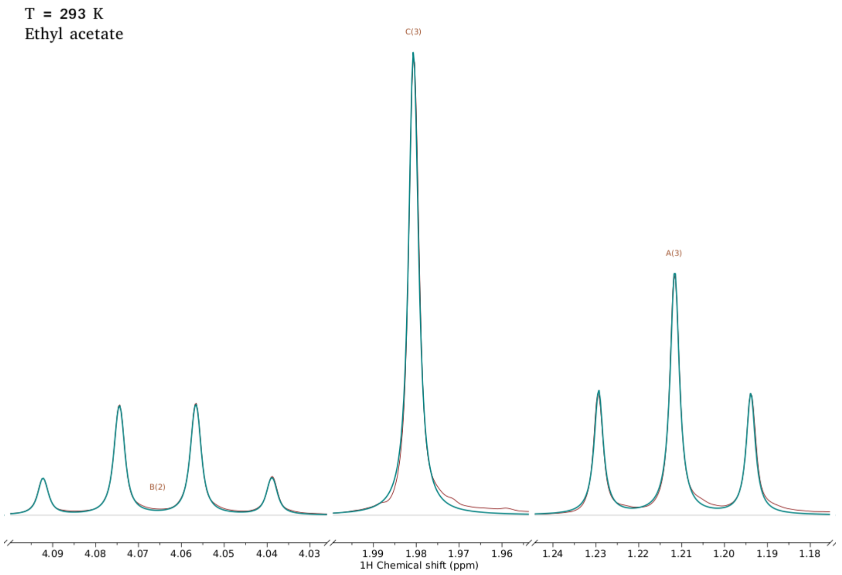

Supplementary Figure 9. Experimental and simulated ^{1}H NMR spectra of EA were obtained at 9.4 T NMR. The samples were prepared as it is described in the text, hydrogenated using an automated system, and then placed into a 5 mm NMR tube for analysis. Simulation NMR parameters for EA: H_A (Triplet, 1.2116 ppm, J(H_A-H_B) =7.12 Hz), H_B (quartet, 4.0654 ppm, J(H_B-H_C) =0.21 Hz,), H_C (singlet, 1.9805 ppm) line width was 0.99 Hz. The temperature of the sample was 293 K.

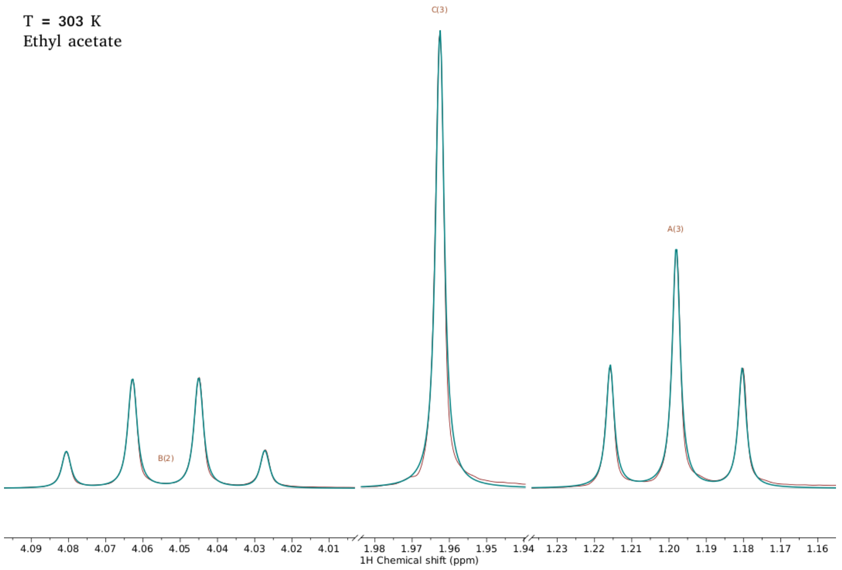

Supplementary Figure 10. Experimental and simulated ^{1}H NMR spectra of EA were obtained at 9.4 T NMR. The samples were prepared as it is described in the text, hydrogenated using an automated system and then placed into a 5 mm NMR tube for the analysis. Simulation NMR parameters for EA: H_A (Triplet, 1.1980 ppm, J (H_A-H_B) =7.12 Hz), H_B (quartet, 4.0538 ppm, J(H_B-H_C) =0.2 Hz), H_C (singlet, 1.9626 ppm) line width was 1 Hz. The temperature of the sample was 303 K.

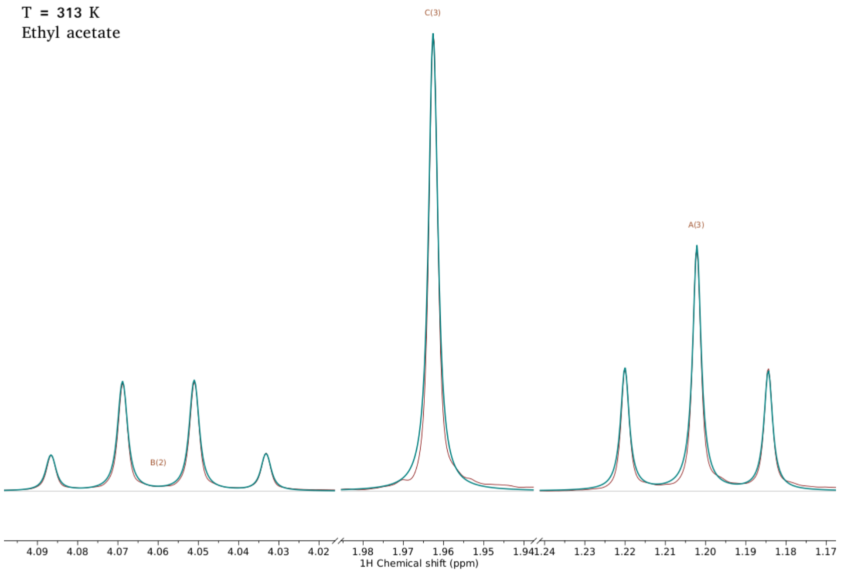

Supplementary Figure 11. Experimental and simulated ^{1}H NMR spectra of EA were obtained at 9.4 T NMR. The samples were prepared as it is described in the text, hydrogenated using an automated system and then placed into a 5 mm NMR tube for the analysis. Simulation NMR parameters for EA: H_A (Triplet, 1.2022 ppm, J (H_A-H_B) =7.13 Hz), H_B (quartet, 4.0598 ppm, J(H_B-H_C) =0.2 Hz), H_C (singlet, 1.9626 ppm) line width was 1 Hz. The temperature of the sample was 313 K.

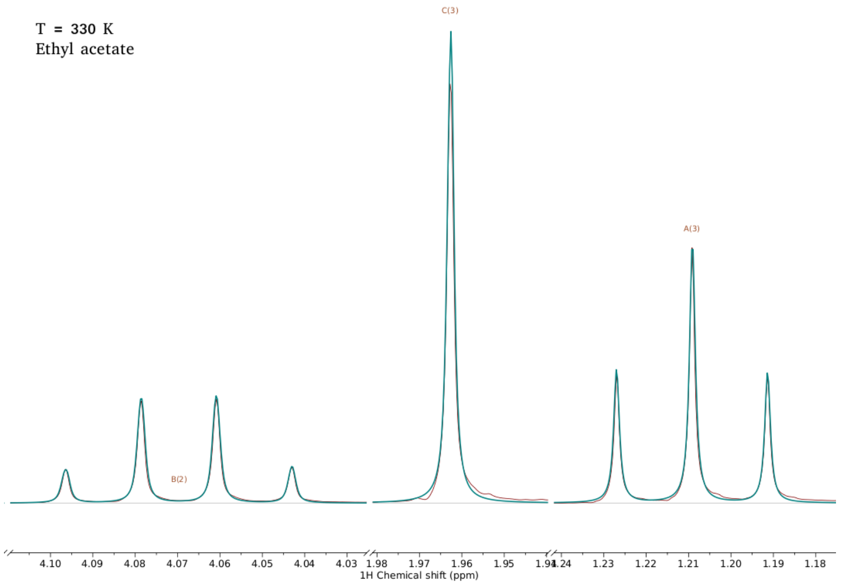

Supplementary Figure 12. Experimental and simulated ^{1}H NMR spectra of EA were obtained at 9.4 T NMR. The samples were prepared as it is described in the text, hydrogenated using an automated system and then placed into a 5 mm NMR tube for the analysis. Simulation NMR parameters for EA: H_A (Triplet, 1.2091 ppm, J (H_A-H_B) =7.12 Hz), H_B (quartet, 4.0696 ppm, J(H_B-H_C) =0.23 Hz), H_C (singlet, 1.9626 ppm) line width was 0.62 Hz. The temperature of the sample was 330 K.

Ethyl pyruvate H_6 (EP)

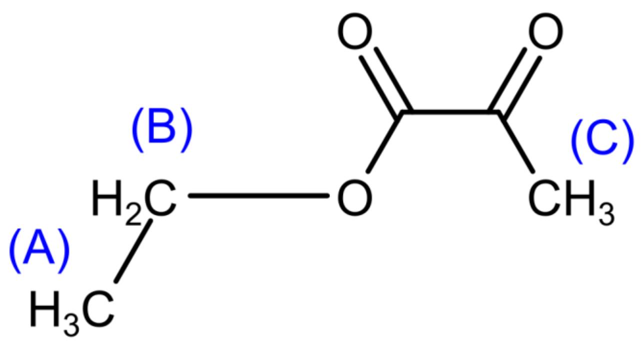

Supplementary Chart 3. Chemical structure of EP.

Supplementary Table 3. Chemical shifts and J coupling constants for EP in acetone were measured at 9.4 T and four temperatures.

T (K)	$\delta(H_A)$ (ppm)	$\delta(H_B)$ (ppm)	$\delta(H_C)$ (ppm)	$\Delta\delta=\delta(H_A)-\delta(H_B)$ (ppm)	$J(H_A-H_B)$ (Hz)	$J(H_B-H_C)$ (Hz)
293	1.2941	4.2501	2.4051	-2.956	7.12	-
303	1.2997	4.2574	2.4036	-2.9577	7.12	0.13
313	1.3052	4.2644	2.4025	-2.9592	7.12	0.11
330	1.3139	4.2755	2.3997	-2.9616	7.12	-

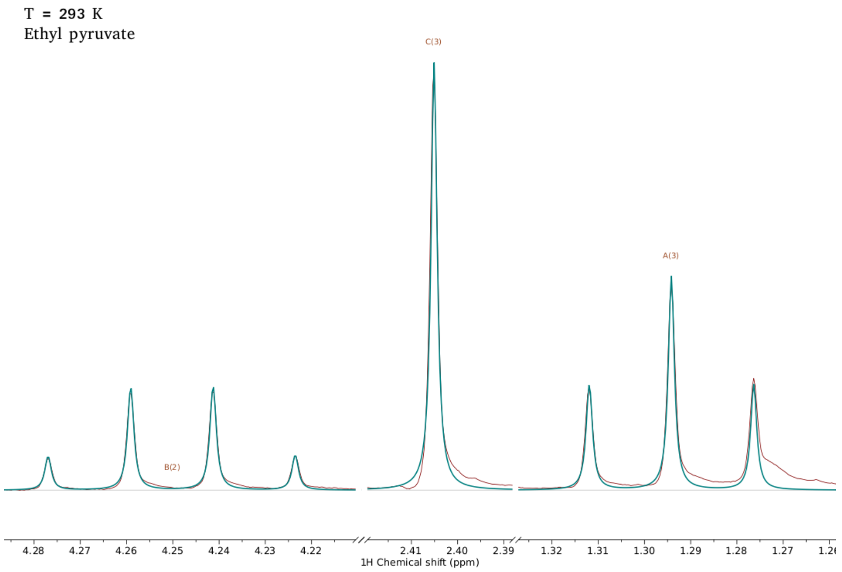

Supplementary Figure 13. Experimental and simulated ^1H NMR spectra of EP obtained at 9.4 T NMR. The samples were prepared as it is described in the text, hydrogenated using an automated system and then placed into a 5 mm NMR tube for the analysis. Simulation NMR parameters for EP: H_A (Triplet, 1.2941 ppm, J (H_A-H_B) =7.12 Hz), H_B (quartet, 4.2501 ppm, J(H_B-H_C) =0 Hz), H_C (singlet, 2.4051 ppm) line width was 0.63 Hz. The temperature of the sample was 293 K.

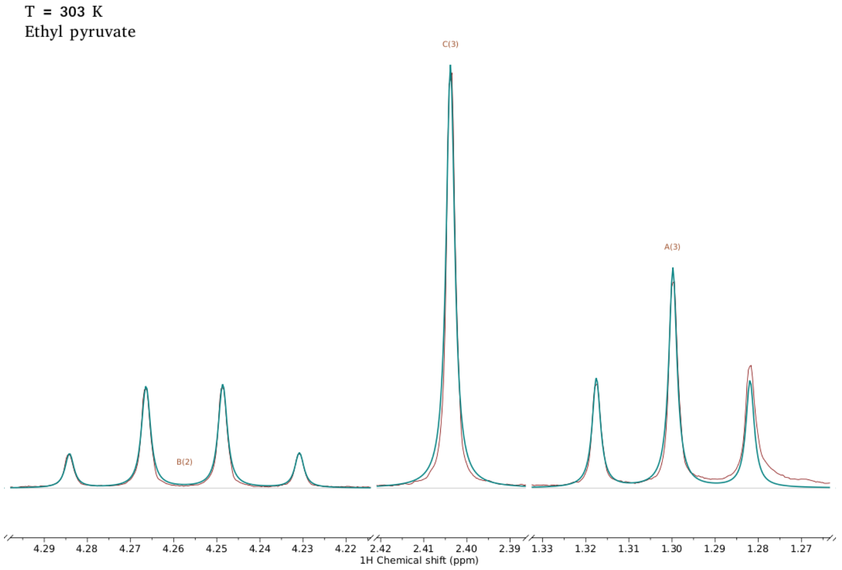

Supplementary Figure 14. Experimental and simulated ^1H NMR spectra of EP obtained at 9.4 T NMR. The samples were prepared as it is described in the text, hydrogenated using an automated system and then placed into a 5 mm NMR tube for the analysis. Simulation NMR parameters for EP: H_A (Triplet, 1.2997 ppm, J (H_A-H_B) =7.12 Hz), H_B (quartet, 4.2574 ppm, J(H_B-H_C) =0.13 Hz), H_C (singlet, 2.4036 ppm) line width was 0.9 Hz. The temperature of the sample was 303 K.

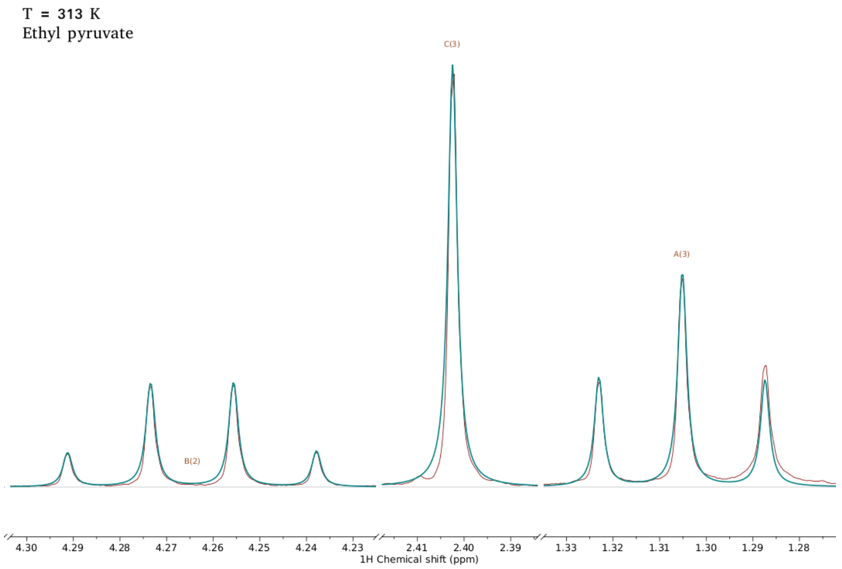

Supplementary Figure 15. Experimental and simulated ^{1}H NMR spectra of EP obtained at 9.4 T NMR. The samples were prepared as it is described in the text, hydrogenated using an automated system, and then placed into a 5 mm NMR tube for analysis. Simulation NMR parameters for EP: H_A (Triplet, 1.3052 ppm, J (H_A-H_B) =7.12 Hz), H_B (quartet, 4.2644 ppm, J(H_B-H_C) =0.2 Hz), H_C (singlet, 2.4025 ppm) line width was 0.9 Hz. The temperature of the sample was 313 K.

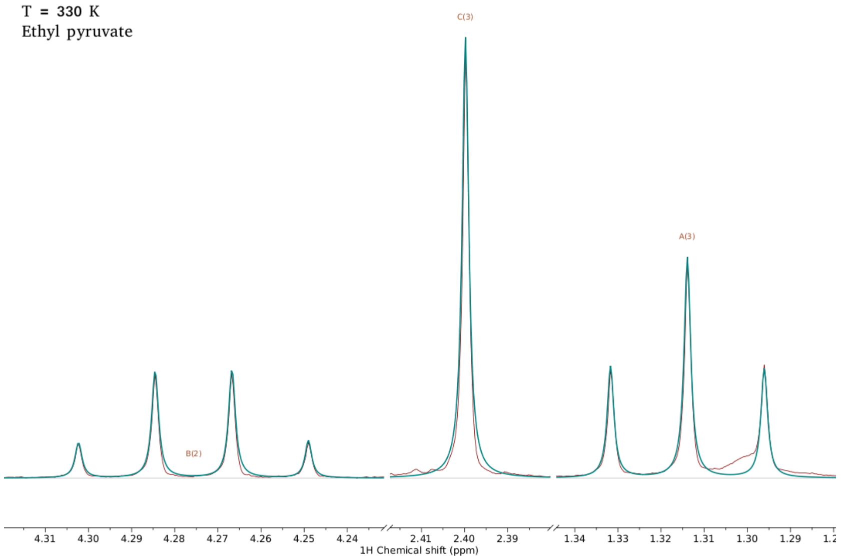

Supplementary Figure 16. Experimental and simulated ^{1}H NMR spectra of EP obtained at 9.4 T NMR. The samples were prepared as it is described in the text, hydrogenated using an automated system, and then placed into a 5 mm NMR tube for the analysis. Simulation NMR parameters for EP: H_A (Triplet, 1.3139 ppm, J (H_A-H_B) =7.12 Hz), H_B (quartet, 4.2755 ppm, J(H_B-H_C) =0 Hz), H_C (singlet, 2.3997 ppm) line width was 0.74 Hz. The temperature of the sample was 330 K.

Supplementary note 6: ESOTHERIC SOT at high and low magnetic fields

Here (**Supplementary Figure 17**), you can see the performance of the ESOTHERIC SOT at 9.4 and 0.57 T magnetic fields with a different number of refocusing pulses.

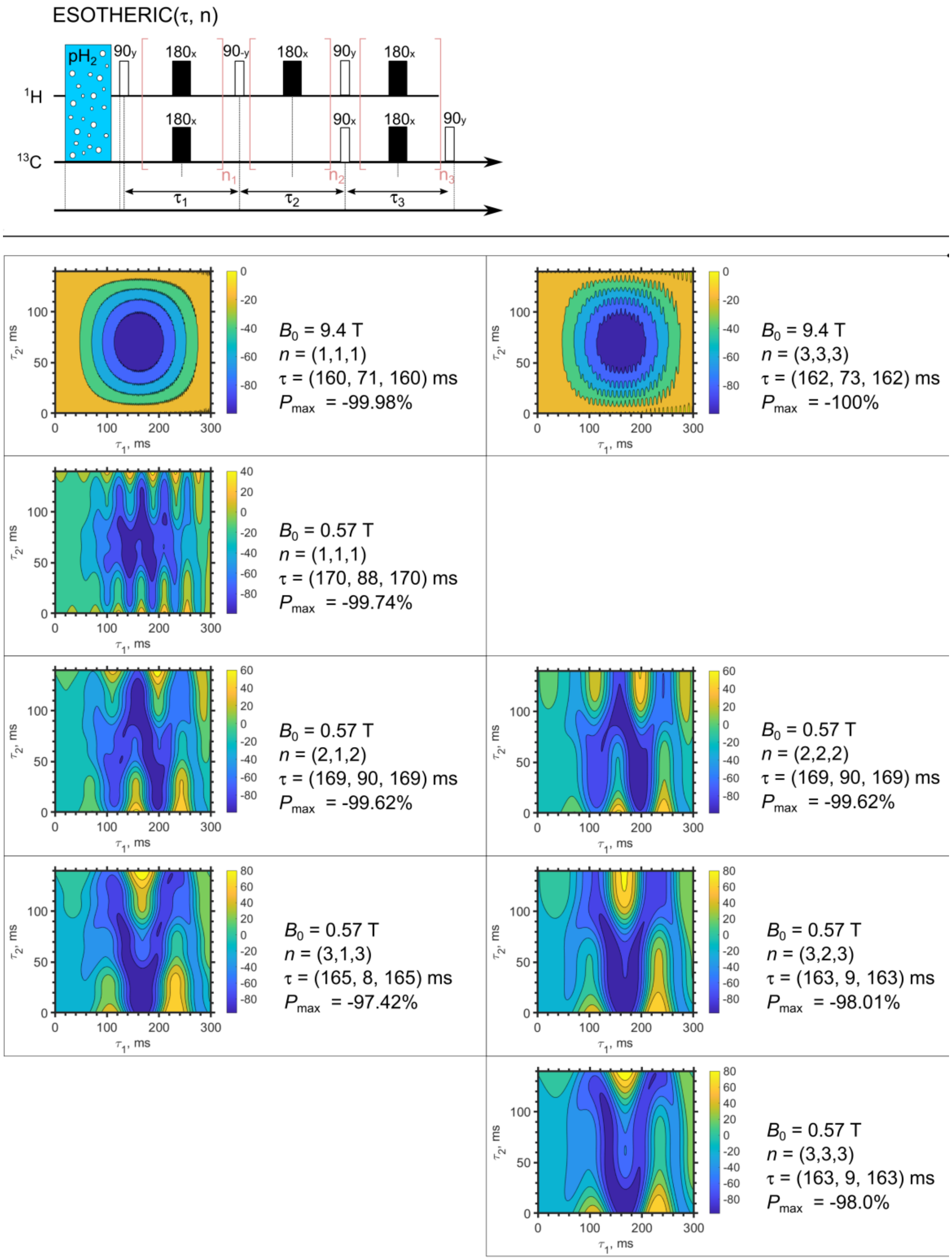

Supplementary Figure 17. The ESOTHERIC SOT and resulting ^{13}C polarization of 1-^{13}C-EP-*d6* at 9.4 and 0.57 T. The ESOTHERIC performance does not change as a function of the number of refocusing pulses when all spins are weakly coupled (B_0 = 9.4 T), while there is a significant effect from the number of refocusing pulses of $n = (n_1, n_2, n_3)$ at B_0 = 0.57 T where protons are intermediately coupled.

Supplementary note 7: ^{13}C Polarization and enhancement

Signal enhancement and polarization were calculated using a 5.3 M ^{13}C-urea sample as a reference as

$$Enhancement = \frac{I(\mathrm{PHIP})}{I(^{13}\mathrm{C}-\mathrm{Urea})} \cdot \frac{[^{13}\mathrm{C}-\mathrm{Urea}]}{[\mathrm{Reagent}]}$$

$$P_{\mathrm{th}} = \tanh\left(\frac{\gamma_{13\mathrm{C}} \cdot \hbar \cdot B_0}{2 \cdot k_{\mathrm{B}} \cdot T}\right)$$

$$P = Enhancement \cdot P_{\mathrm{th}}$$

where $I(\mathrm{PHIP})$ is the total integral for the PHIP spectrum after SOT, $I(^{13}\mathrm{C}-\mathrm{Urea})$ is the integral for the ^{13}C-urea sample at thermal equilibrium, [X] are concentrations of urea or a reagent (VP-*d6* or VA-*d6*, 50 mM), P_{th} is the value of thermal polarization, and P is the resulting ^{13}C polarization.
